# Supplementary material for: Phagocytic glia are obligatory intermediates in transmission of mutant huntingtin aggregates across neuronal synapses
Source: eLife. 2020 May 28;9:e58499. doi: 10.7554/eLife.58499 (PMC7297539; doi:10.7554/eLife.58499)
Supplement: Supplementary file 1. [file elife-58499-supp1.docx]

**Supplementary file 1. Full genotypes of flies used in this study**

| **Figure** | **Full Genotype** |
| --- | --- |
| **Figure 1** | |
| C | *Or67d-QF,UAS-Htt_ex1_Q25-GFP^attP3^/(+/Y) ; GH146-Gal4/QUAS-Htt_ex1_Q25-mCherry^attP24^* |
| D | *Or67d-QF,UAS-Htt_ex1_Q25-GFP^attP3^/(+/Y) ; GH146-Gal4/QUAS-Htt_ex1_Q91-mCherry^attP24^* |
| E,F,G,J,K | *Or67d-QF,UAS-Htt_ex1_Q25-GFP^attP3^/(+/Y) ; GH146-Gal4,QUAS-Htt_ex1_Q91-mCherry^attP24^/+* |
| H,I,J,K | *Or67d-QF,UAS-Htt_ex1_Q25-GFP^attP3^/QUAS-RFP-Htt_ex1-12_Q138^attP3B^ ; GH146-Gal4/+* |
| **Figure 1 - figure supplement 1** | |
| A,F | *elav[C155]-Gal4/UAS-Htt_ex1_Q91-mCherry^attP3^* |
| B,F | *elav[C155]-Gal4/+ ; UAS-Htt_ex1_Q25-GFP^attP24^/+* |
| C,F | *elav[C155]-Gal4/UAS-Htt_ex1_Q91-mCherry^attP3^ ; UAS-Htt_ex1_Q25-GFP^attP24^/+* |
| D,F | *elav[C155]-Gal4/UAS-Htt_ex1_Q91-mCherry^attP3^ ; UAS-mCD8-GFP/+* |
| E,F | *elav[C155]-Gal4/UAS-Htt_ex1_Q91-mCherry^attP3^ ; UAS-GFP^attP24^/+* |
| **Figure 1 - figure supplement 2** | |
| A,B,C,D | *Or67d-QF,UAS-Htt_ex1_Q25-GFP^attP3^/Y ; GH146-Gal4,QUAS-Htt_ex1_Q91-mCherry^attP24^/+* |
| **Figure 1 - figure supplement 3** | |
| A,E,F | *Or67d-QF,UAS-Htt_ex1_Q25-GFP^attP3^/+ ; GH146-Gal4/QUAS-Htt_ex1_Q25-mCherry^attP24^* |
| B,E,F | *Or67d-QF/Y ; GH146-Gal4,QUAS-Htt_ex1_Q91-mCherry^attP24^/UAS-mCD8-GFP* |
| C,E,F | *Or67d-QF,UAS-Htt_ex1_Q25-GFP^attP3^/QUAS-Gal80 ; GH146-Gal4/QUAS-Htt_ex1_Q91-mCherry^attP24^* |
| D,E,F | *Or67d-QF,UAS-Htt_ex1_Q25-GFP^attP3^/+ ; GH146-Gal4/QUAS-Htt_ex1_Q91-mCherry^attP24^ ; UAS-QS/+* |
| **Figure 1 - figure supplement 4** | |
| A1-4,B1-4,C,D | *pebbled-Gal4/QUAS-Htt_ex1_Q91-mCherry^attP3^ ; UAS-Htt_ex1_Q25-GFP^attP24^/+ ; GH146-QF/+* |
| **Figure 2** | |
| A,B,E,F,G | *Or67d-QF,UAS-Htt_ex1_Q25-GFP^attP3^/(+/Y) ; GH146-Gal4,QUAS-Htt_ex1_Q91-mCherry^attP24^/+* |
| C,D,F,H | *Or67d-QF,UAS-Htt_ex1_Q25-GFP^attP3^/QUAS-RFP-Htt_ex1-12_Q138^attP3B^ ; GH146-Gal4/+* |
| **Figure 3** | |
| A,C,D | *Or67d-QF,UAS-Htt_ex1_Q25-GFP^attP3^/Y ; GH146-Gal4,QUAS-Htt_ex1_Q91-mCherry^attP24^/+ ; QUAS-nucLacZ/+* |
| B,C,D | *Or67d-QF,UAS-Htt_ex1_Q25-GFP^attP3^/Y ; GH146-Gal4,QUAS-Htt_ex1_Q91-mCherry^attP24^/+; QUAS-shibire^ts1^#5/+* |
| C,D | *Or67d-QF,UAS-Htt_ex1_Q25-GFP^attP3^/Y ; GH146-Gal4,QUAS-Htt_ex1_Q91-mCherry^attP24^/+; QUAS-shibire^ts1^#7/+* |
| E,G,H | *Or67d-QF,UAS-Htt_ex1_Q25-GFP^attP3^/Y ; GH146-Gal4,QUAS-Htt_ex1_Q91-mCherry^attP24^/+ ; QUAS-nucLacZ/+* |
| F,G,H | *Or67d-QF,UAS-Htt_ex1_Q25-GFP^attP3^/Y ; GH146-Gal4,QUAS-Htt_ex1_Q91-mCherry^attP24^/QUAS-TeTxLC#4c ; +/(Dh/Tm6b)* |
| G,H | *Or67d-QF,UAS-Htt_ex1_Q25-GFP^attP3^/Y ; GH146-Gal4,QUAS-Htt_ex1_Q91-mCherry^attP24^/Pin; QUAS-TeTxLC#9c/+* |
| I,K,L | *Or67d-QF,UAS-Htt_ex1_Q25-GFP^attP3^/+ ; GH146-Gal4,QUAS-Htt_ex1_Q91-mCherry^attP24^/+ ; QUAS-nucLacZ/+* |
| K,L | *Or67d-QF,UAS-Htt_ex1_Q25-GFP^attP3^/QUAS-dTrpA#5 ; GH146-Gal4,QUAS-Htt_ex1_Q91-mCherry^attP24^/+* |
| J,K,L | *Or67d-QF,UAS-Htt_ex1_Q25-GFP^attP3^/+ ; GH146-Gal4,QUAS-Htt_ex1_Q91-mCherry^attP24^/+ ; QUAS-dTrpA#6/+* |
| K,L | *Or67d-QF,UAS-Htt_ex1_Q25-GFP^attP3^/+ ; GH146-Gal4,QUAS-Htt_ex1_Q91-mCherry^attP24^/QUAS-dTrpA#7* |
| **Figure 4** | |
| A,C | *Or67d-QF,QUAS-Htt_ex1_Q91-mCherry^attP3^/+ ; QUAS-mCD8-GFP/+ ; QUAS-nucLacZ/+* |
| B,C | *Or67d-QF,QUAS-Htt_ex1_Q91-mCherry^attP3^/+ ; QUAS-mCD8-GFP/+ ; QUAS-shibire^ts1^#7/+* |
| D,F,G | *Or67d-QF,QUAS-Htt_ex1_Q91-mCherry^attP3^/Y ; UAS-Htt_ex1_Q25-YFP^attP24^/+ ; repo-Gal4/QUAS-nucLacZ* |
| E,F,G | *Or67d-QF,QUAS-Htt_ex1_Q91-mCherry^attP3^/Y ; UAS-Htt_ex1_Q25-YFP^attP24^/+ ; repo-Gal4/QUAS-shibire^ts1^#7* |
| F,G | *Or67d-QF,QUAS-Htt_ex1_Q91-mCherry^attP3^/Y ; UAS-Htt_ex1_Q25-YFP^attP24^/+ ; repo-Gal4/QUAS-shibire^ts1^#5* |
| **Figure 5** | |
| A,C,D | *Or67d-QF,UAS-Htt_ex1_Q25-GFP^attP3^/(+/Y) ; GH146-Gal4,QUAS-Htt_ex1_Q91-mCherry^attP24^/+ ; drpr^Δ5^/+* |
| B,C,D | *Or67d-QF,UAS-Htt_ex1_Q25-GFP^attP3^/(+/Y) ; GH146-Gal4,QUAS-Htt_ex1_Q91-mCherry^attP24^/+ ; drpr^Δ5^/drpr^Δ5^* |
| E,G | *Or67d-QF,QUAS-Htt_ex1_Q91-mCherry^attP3^/(+/Y)* |
| F,G | *Or67d-QF,QUAS-Htt_ex1_Q91-mCherry^attP3^/(+/Y) ; ; drpr^Δ5^/drpr^Δ5^* |
| **Figure 5 - figure supplement 1** | |
| A,C,D | *Or67d-QF,UAS-Htt_ex1_Q25-GFP^attP3^/QUAS-shibire^ts1^#2 ; GH146-Gal4,QUAS-Htt_ex1_Q91-mCherry^attP24^/+* |
| B,C,D | *Or67d-QF,UAS-Htt_ex1_Q25-GFP^attP3^/QUAS-shibire^ts1^#2 ; GH146-Gal4,QUAS-Htt_ex1_Q91-mCherry^attP24^/+ ; drpr^Δ5^/drpr^Δ5^* |
| **Figure 5 - figure supplement 2** | |
| A,E,F | *Or67d-QF,UAS-Htt_ex1_Q25-GFP^attP3^/Y ; GH146-Gal4,QUAS-Htt_ex1_Q91-mCherry^attP24^/+* |
| B,E,F | *Or67d-QF,UAS-Htt_ex1_Q25-GFP^attP3^/Y ; GH146-Gal4,QUAS-Htt_ex1_Q91-mCherry^attP24^/repo-Gal80* |
| C,E,F | *Or67d-QF,UAS-Htt_ex1_Q25-GFP^attP3^/Y ; GH146-Gal4,QUAS-Htt_ex1_Q91-mCherry^attP24^/+ ; UAS-FFLuc ^attP2^/+* |
| D,E,F | *Or67d-QF,UAS-Htt_ex1_Q25-GFP^attP3^/Y ; GH146-Gal4,QUAS-Htt_ex1_Q91-mCherry^attP24^/+ ; UAS-Draper^RNAi#5^/+* |
| **Figure 5 - figure supplement 3** | |
| A1-2,C,E,F | *Or67d-QF,QUAS-Htt_ex1_Q91-mCherry^attP3^/+ ; UAS-Atg8a-GFP/+ ; repo-Gal4/+* |
| B1-2,D,E,F | *Or67d-QF,QUAS-Htt_ex1_Q91-mCherry^attP3^/+ ; UAS-GFP-Lamp1/+ ; repo-Gal4/+* |
| E,F | *Or67d-QF,QUAS-Htt_ex1_Q91-mCherry^attP3^/+ ; UAS-mCD8-GFP/+ ; repo-Gal4/+* |
| **Figure 6** | |
| A,E | *Or83b-Gal4,UAS-Htt_ex1_Q25-GFP^attP24^/+ ; drpr^Δ5^/+* |
| B,E | *Or83b-Gal4,UAS-Htt_ex1_Q91-GFP^attP24^/+ ; drpr^Δ5^/+* |
| C,E | *Or83b-Gal4,UAS-Htt_ex1_Q25-GFP^attP24^/+ ; drpr^Δ5^/drpr^Δ5^* |
| D,E | *Or83b-Gal4,UAS-Htt_ex1_Q91-GFP^attP24^/+ ; drpr^Δ5^/drpr^Δ5^* |
| F,J,K | *Or67d-QF,UAS-Htt_ex1_Q25-GFP^attP3^/Y ; GH146-Gal4,QUAS-Htt_ex1_Q91-mCherry^attP24^/+ ; QUAS-nucLacZ/+* |
| G,J,K | *Or67d-QF,UAS-Htt_ex1_Q25-GFP^attP3^/Y ; GH146-Gal4,QUAS-Htt_ex1_Q91-mCherry^attP24^/+; QUAS-p35/+* |
| H,J,K | *Or67d-QF,UAS-Htt_ex1_Q25-GFP^attP3^/Y ; GH146-Gal4,QUAS-Htt_ex1_Q91-mCherry^attP24^/UAS-LacZ* |
| I,J,K | *Or67d-QF,UAS-Htt_ex1_Q25-GFP^attP3^/Y ; GH146-Gal4,QUAS-Htt_ex1_Q91-mCherry^attP24^/UAS-p35* |
| **Figure 7** | |
| B,C,D,E,F,G,I,J | *Or67d-QF,QUAS-Htt_ex1_Q91-mCherry^attP3^/+ ; GH146-LexA::GAD,LexAop-Htt_ex1_Q25-YFP^attP24^/UAS-Htt_ex1_Q25-3xHA^attP24^ ; repo-Gal4/+* |
| H,I,J | *Or67d-QF,QUAS-Htt_ex1_Q91-mCherry^attP3^/+ ; GH146-LexA::GAD,LexAop-Htt_ex1_Q25-YFP^attP24^/UAS-Htt_ex1_Q25-3xHA^attP24^ ; repo-Gal4/UAS-Draper^RNAi#5^* |
| **Figure 7 - figure supplement 1** | |
| A1-2 | *GH146-LexA::GAD,LexAop-Htt_ex1_Q25-YFP^attP24^/UAS-Htt_ex1_Q25-3xHA^attP24^ ; repo-Gal4/+* |
| B1-2 | *Or67d-QF/+ ; GH146-LexA::GAD,LexAop-Htt_ex1_Q25-YFP^attP24^/QUAS-Htt_ex1_Q25-mCherry^attP24^* |
| C1-2 | *Or67d-QF/+ ; QUAS-Htt_ex1_Q25-mCherry^attP24^/UAS-Htt_ex1_Q25-3xHA^attP24^ ; repo-Gal4/+* |
| **Video 1** | |
|  | *Or67d-QF,UAS-Htt_ex1_Q25-GFP^attP3^/(+/Y) ; GH146-Gal4,QUAS-Htt_ex1_Q91-mCherry^attP24^/+* |
