# Supplementary material for: Phagocytic glia are obligatory intermediates in transmission of mutant huntingtin aggregates across neuronal synapses
Source: eLife. 2020 May 28;9:e58499. doi: 10.7554/eLife.58499 (PMC7297539; doi:10.7554/eLife.58499)
Supplement: Supplementary file 2. — Symbols and colors in ‘Significance’ column match those shown in figures. [file elife-58499-supp2.docx]

**Supplementary file 2. Sample sizes and statistical analyses used in this study.** Symbols and colors in “Significance” column match those shown in figures.

| **Figure** | **Sample** | | **Sample size (n)** | **Statistical Test** | **Comparisons** | | **P-value/**  **Adj P-value** | **Significance** |
| --- | --- | --- | --- | --- | --- | --- | --- | --- |
| **Figure 1** | | | | | | | | |
| Fig. 1J | **Htt_ex1_ males** | | **1d (14), 7d (15), 14d (20), 21d (14)** | Two way ANOVA | - 1. Htt_ex1_ males (1d, 7d, 14d, 21d)   2. Htt_ex1_ females (1d, 7d, 14d, 21d)   3. Htt_ex1-12_ females (1d, 7d, 14d, 21d) | | Row (age): <0.0001 | **** |
|  |  |  |  |  |  |  | Column (genotype): <0.0001 | **** |
|  | **Htt_ex1_ females** | | **1d (10), 7d (10), 14d (17), 21d (18)** | Tukey’s multiple comparisons | Htt_ex1_ males (1d), Htt_ex1_ males (7d) | | <0.0001 | **** |
|  | **Htt_ex1-12_ females** | | **1d (10), 7d (12), 14d (14), 21d (20)** |  | Htt_ex1_ males (1d), Htt_ex1_ males (14d) | | <0.0001 | **** |
|  |  | | |  | Htt_ex1_ males (1d), Htt_ex1_ males (21d) | | <0.0001 | **** |
|  |  |  |  |  | Htt_ex1_ males (7d), Htt_ex1_ males (14d) | | 0.2204 | n.s. |
|  |  |  |  |  | Htt_ex1_ males (7d), Htt_ex1_ males (21d) | | <0.0001 | **** |
|  |  |  |  |  | Htt_ex1_ males (14d), Htt_ex1_ males (21d) | | 0.0083 | ** |
|  |  |  |  |  | Htt_ex1_ females (1d), Htt_ex1_ females (7d) | | 0.0219 | * |
|  |  |  |  |  | Htt_ex1_ females (1d), Htt_ex1_ females (14d) | | <0.0001 | **** |
|  |  |  |  |  | Htt_ex1_ females (1d), Htt_ex1_ females (21d) | | <0.0001 | **** |
|  |  |  |  |  | Htt_ex1_ females (7d), Htt_ex1_ females (14d) | | 0.0006 | **­­* |
|  |  |  |  |  | Htt_ex1_ females (7d), Htt_ex1_ females (21d) | | <0.0001 | **** |
|  |  |  |  |  | Htt_ex1_ females (14d), Htt_ex1_ females (21d) | | 0.6611 | n.s. |
|  |  |  |  |  | Htt_ex1-12_ females (1d), Htt_ex1-12_ females (7d) | | 0.8839 | n.s. |
|  |  |  |  |  | Htt_ex1-12_ females (1d), Htt_ex1-12_ females (14d) | | 0.0084 | ** |
|  |  |  |  |  | Htt_ex1-12_ females (1d), Htt_ex1-12_ females (21d) | | 0.0001 | *** |
|  |  |  |  |  | Htt_ex1-12_ females (7d), Htt_ex1-12_ females (14d) | | 0.0507 | n.s. |
|  |  |  |  |  | Htt_ex1-12_ females (7d), Htt_ex1-12_ females (21d) | | 0.0014 | ** |
|  |  |  |  |  | Htt_ex1-12_ females (14d), Htt_ex1-12_ females (21d) | | 0.7439 | n.s. |
|  |  |  |  |  | Htt_ex1_ males, Htt_ex1_ females (1d) | | 0.0432 | # |
|  |  |  |  |  | Htt_ex1_ males, Htt_ex1_ females (7d) | | <0.0001 | #### |
|  |  |  |  |  | Htt_ex1_ males, Htt_ex1_ females (14d) | | <0.0001 | #### |
|  |  |  |  |  | Htt_ex1_ males, Htt_ex1_ females (21d) | | <0.0001 | #### |
|  |  |  |  |  | Htt_ex1_ females, Htt_ex1-12_ females (1d) | | 0.9953 | n.s. |
|  |  |  |  |  | Htt_ex1_ females, Htt_ex1-12_ females (7d) | | 0.0467 | # |
|  |  |  |  |  | Htt_ex1_ females, Htt_ex1-12_ females (14d) | | <0.0001 | #### |
|  |  |  |  |  | Htt_ex1_ females, Htt_ex1-12_ females (21d) | | <0.0001 | #### |
| Fig. 1K | **Htt_ex1_ males** | **1d (14), 7d (15), 14d (20), 21d (14)** | | Two way ANOVA | - 1. Htt_ex1_ males (1d, 7d, 14d, 21d)   5-8. Htt_ex1_ females (1d, 7d, 14d, 21d)   - 1. Htt_ex1-12_ females (1d, 7d, 14d, 21d) | | Row (age): <0.0001 | **** |
|  |  |  |  |  |  |  | Column (genotype): 0.0126 | * |
|  | **Htt_ex1_ females** | **1d (10), 7d (10), 14d (17), 21d (18)** | | Tukey’s multiple comparisons | Htt_ex1_ males (1d), Htt_ex1_ males (7d) | | 0.0025 | ** |
|  | **Htt_ex1-12_ females** | **1d (10), 7d (12), 14d (14), 21d (20)** | |  | Htt_ex1_ males (1d), Htt_ex1_ males (14d) | | <0.0001 | **** |
|  |  | | |  | Htt_ex1_ males (1d), Htt_ex1_ males (21d) | | <0.0001 | **** |
|  |  |  |  |  | Htt_ex1_ males (7d), Htt_ex1_ males (14d) | | 0.7737 | n.s. |
|  |  |  |  |  | Htt_ex1_ males (7d), Htt_ex1_ males (21d) | | 0.0005 | *** |
|  |  |  |  |  | Htt_ex1_ males (14d), Htt_ex1_ males (21d) | | 0.0053 | ** |
|  |  |  |  |  | Htt_ex1_ females (1d), Htt_ex1_ females (7d) | | 0.9468 | n.s. |
|  |  |  |  |  | Htt_ex1_ females (1d), Htt_ex1_ females (14d) | | 0.0322 | * |
|  |  |  |  |  | Htt_ex1_ females (1d), Htt_ex1_ females (21d) | | <0.0001 | **** |
|  |  |  |  |  | Htt_ex1_ females (7d), Htt_ex1_ females (14d) | | 0.1431 | n.s. |
|  |  |  |  |  | Htt_ex1_ females (7d), Htt_ex1_ females (21d) | | <0.0001 | **** |
|  |  |  |  |  | Htt_ex1_ females (14d), Htt_ex1_ females (21d) | | <0.0001 | **** |
|  |  |  |  |  | Htt_ex1-12_ females (1d), Htt_ex1-12_ females (7d) | | 0.9236 | n.s. |
|  |  |  |  |  | Htt_ex1-12_ females (1d), Htt_ex1-12_ females (14d) | | 0.0127 | * |
|  |  |  |  |  | Htt_ex1-12_ females (1d), Htt_ex1-12_ females (21d) | | <0.0001 | **** |
|  |  |  |  |  | Htt_ex1-12_ females (7d), Htt_ex1-12_ females (14d) | | 0.0541 | n.s. |
|  |  |  |  |  | Htt_ex1-12_ females (7d), Htt_ex1-12_ females (21d) | | <0.0001 | **** |
|  |  |  |  |  | Htt_ex1-12_ females (14d), Htt_ex1-12_ females (21d) | | 0.1119 | n.s. |
|  |  |  |  |  | Htt_ex1_ males, Htt_ex1_ females (1d) | | 0.9543 | n.s. |
|  |  |  |  |  | Htt_ex1_ males, Htt_ex1_ females (7d) | | 0.0495 | # |
|  |  |  |  |  | Htt_ex1_ males, Htt_ex1_ females (14d) | | 0.3798 | n.s. |
|  |  |  |  |  | Htt_ex1_ males, Htt_ex1_ females (21d) | | 0.2336 | n.s. |
|  |  |  |  |  | Htt_ex1_ females, Htt_ex1-12_ females (1d) | | 0.9594 | n.s. |
|  |  |  |  |  | Htt_ex1_ females, Htt_ex1-12_ females (7d) | | 0.9704 | n.s. |
|  |  |  |  |  | Htt_ex1_ females, Htt_ex1-12_ females (14d) | | 0.9878 | n.s. |
|  |  |  |  |  | Htt_ex1_ females, Htt_ex1-12_ females (21d) | | 0.0001 | ### |
| **Figure 1 - figure supplement 1** | | | | | | | | |
| Fig. 1 - fig supp 1F | **Htt_ex1_Q91** | | **8** | One way ANOVA | 1. Htt_ex1_Q91 2. Htt_ex1_Q25 3. Htt_ex1_Q91 + Htt_ex1_Q25 4. Htt_ex1_Q91 + mCD8 5. Htt_ex1_Q91 + GFP | | <0.0001 | **** |
|  | **Htt_ex1_Q25** | | **6** | Tukey’s multiple comparisons | Htt_ex1_Q91; Htt_ex1_Q91 + Htt_ex1_Q25 | | <0.0001 | **** |
|  | **Htt_ex1_Q91 + Htt_ex1_Q25** | | **6** |  | Htt_ex1_Q25; Htt_ex1_Q91 + Htt_ex1_Q25 | | <0.0001 | **** |
|  | **Htt_ex1_Q91 + mCD8** | | **6** |  | Htt_ex1_Q91 + Htt_ex1_Q25; Htt_ex1_Q91 + mCD8 | | <0.0001 | **** |
|  | **Htt_ex1_Q91 + GFP** | | **7** |  | Htt_ex1_Q91 + Htt_ex1_Q25; Htt_ex1_Q91 + GFP | | <0.0001 | **** |
| **Figure 1 - figure supplement 2** | | | | | | | | |
| Fig. 1 – fig supp 2D |  | | **14** | One way ANOVA | 1. manual (A) 2. seg: Htt_ex1_Q91>Htt_ex1_Q25 (B1) 3. seg: Htt_ex1_Q25>Htt_ex1_Q91 (B2) | | 0.5582 | n.s. |
|  |  | | | Tukey’s multiple comparisons | manual (A), seg: Htt_ex1_Q91>Htt_ex1_Q25 (B1) | | 0.6262 | n.s. |
|  |  |  |  |  | manual (A), seg: Htt_ex1_Q25>Htt_ex1_Q91 (B2) | | 0.6089 | n.s. |
|  |  |  |  |  | seg: Htt_ex1_Q91>Htt_ex1_Q25 (B1), seg: Htt_ex1_Q25>Htt_ex1_Q91 (B2) | | 0.9996 | n.s. |
| **Figure 1 - figure supplement 3** | | | | | | | | |
| Fig. 1 – fig supp 3E | **Htt_ex1_Q25 in ORNs and Htt_ex1_Q25 in PNs** | | **6** | One way ANOVA | 1. Htt_ex1_Q25 in ORNs and Htt_ex1_Q25 in PNs 2. Htt_ex1_Q91 in ORNs and mCD8 in PNs 3. Htt_ex1_Q91 + Gal80 in ORNs and Htt_ex1_Q25 in PNs 4. Htt_ex1_Q91 in ORNs and Htt_ex1_Q25 + QS in PNs | | <0.0001 | **** |
|  | **Htt_ex1_Q91 in ORNs and mCD8 in PNs** | | **8** | Tukey’s multiple comparisons | Htt_ex1_Q25 in ORNs and Htt_ex1_Q25 in PNs,  Htt_ex1_Q91 in ORNs and mCD8 in PNs | | <0.0001 | **** |
|  | **Htt_ex1_Q91 + Gal80 in ORNs and Htt_ex1_Q25 in PNs** | | **6** |  | Htt_ex1_Q25 in ORNs and Htt_ex1_Q25 in PNs,  Htt_ex1_Q91 + Gal80 in ORNs and Htt_ex1_Q25 in PNs | | <0.0001 | **** |
|  | **Htt_ex1_Q91 in ORNs and Htt_ex1_Q25 + QS in PNs** | | **6** |  | Htt_ex1_Q25 in ORNs and Htt_ex1_Q25 in PNs,  Htt_ex1_Q91 in ORNs and Htt_ex1_Q25 + QS in PNs | | <0.0001 | **** |
|  |  | | |  | Htt_ex1_Q91 in ORNs and mCD8 in PNs,  Htt_ex1_Q91 + Gal80 in ORNs and Htt_ex1_Q25 in PNs | | 0.2281 | n.s. |
|  |  |  |  |  | Htt_ex1_Q91 in ORNs and mCD8 in PNs,  Htt_ex1_Q91 in ORNs and Htt_ex1_Q25 + QS in PNs | | 0.9824 | n.s. |
|  |  |  |  |  | Htt_ex1_Q91 + Gal80 in ORNs and Htt_ex1_Q25 in PNs,  Htt_ex1_Q91 in ORNs and Htt_ex1_Q25 + QS in PNs | | 0.1568 | n.s. |
| Fig. 1 – fig supp 3F | **Htt_ex1_Q25 in ORNs and Htt_ex1_Q25 in PNs** | | **6** | One way ANOVA | 1. Htt_ex1_Q25 in ORNs and Htt_ex1_Q25 in PNs 2. Htt_ex1_Q91 in ORNs and mCD8 in PNs 3. Htt_ex1_Q91 + Gal80 in ORNs and Htt_ex1_Q25 in PNs 4. Htt_ex1_Q91 in ORNs and Htt_ex1_Q25 + QS in PNs | | 0.0011 | ** |
|  | **Htt_ex1_Q91 in ORNs and mCD8 in PNs** | | **8** | Tukey’s multiple comparisons | Htt_ex1_Q25 in ORNs and Htt_ex1_Q25 in PNs,  Htt_ex1_Q91 in ORNs and mCD8 in PNs | | >0.9999 | n.s. |
|  | **Htt_ex1_Q91 + Gal80 in ORNs and Htt_ex1_Q25 in PNs** | | **6** |  | Htt_ex1_Q25 in ORNs and Htt_ex1_Q25 in PNs,  Htt_ex1_Q91 + Gal80 in ORNs and Htt_ex1_Q25 in PNs | | 0.0078 | ** |
|  | **Htt_ex1_Q91 in ORNs and Htt_ex1_Q25 + QS in PNs** | | **6** |  | Htt_ex1_Q25 in ORNs and Htt_ex1_Q25 in PNs,  Htt_ex1_Q91 in ORNs and Htt_ex1_Q25 + QS in PNs | | 0.0110 | ** |
|  |  | | |  | Htt_ex1_Q91 in ORNs and mCD8 in PNs,  Htt_ex1_Q91 + Gal80 in ORNs and Htt_ex1_Q25 in PNs | | 0.0043 | ** |
|  |  |  |  |  | Htt_ex1_Q91 in ORNs and mCD8 in PNs,  Htt_ex1_Q91 in ORNs and Htt_ex1_Q25 + QS in PNs | | 0.0063 | ** |
|  |  |  |  |  | Htt_ex1_Q91 + Gal80 in ORNs and Htt_ex1_Q25 in PNs,  Htt_ex1_Q91 in ORNs and Htt_ex1_Q25 + QS in PNs | | 0.9987 | n.s. |
| **Figure 1 - figure supplement 4** | | | | | | | | |
| Fig. 1 – fig supp 4C |  | | **7d (5), 14d (5)** | One way ANOVA | 1. 1d 2. 7d 3. 14d | | 0.1203 | n.s. |
|  |  | | | Tukey’s multiple comparisons | 1d, 7d | | 0.4402 | n.s. |
|  |  |  |  |  | 1d, 14d | | 0.1027 | n.s. |
|  |  |  |  |  | 7d, 14d | | 0.6190 | n.s. |
| Fig. 1 – fig supp 4D |  | | **7d (5), 14d (5)** | One way ANOVA | 1. 1d 2. 7d 3. 14d | | 0.6936 | n.s. |
|  |  | | | Tukey’s multiple comparisons | 1d, 7d | | 0.6691 | n.s. |
|  |  |  |  |  | 1d, 14d | | 0.9025 | n.s. |
|  |  |  |  |  | 7d, 14d | | 0.9025 | n.s. |
| **Figure 2** | | | | | | | | |
| Fig. 2E3 | **all** | | **28** | n/a | | | | |
| Fig. 2F | **Htt_ex1_Q91** | | **1d (1,138), 7d (2,235), 14d (4309), 21d (5,435)** | One way ANOVA | - 1. Htt_ex1_Q91 (1d, 7d, 14d, 21d)   2. Htt_ex1_Q91+Htt_ex1_Q25 (1d, 7d, 14d, 21d)   3. Htt_ex1-12_Q138 (1d, 7d, 14d, 21d)   4. Htt_ex1-12_Q138+Htt_ex1_Q25 (1d, 7d, 14d, 21d) | | <0.0001 | **** |
|  | **Htt_ex1_Q91+Htt_ex1_Q25** | | **1d (8), 7d (36), 14d (319), 21d (392)** | Tukey’s multiple comparisons | Htt_ex1_Q91 (1d), Htt_ex1_Q91 (7d) | | <0.0001 | **** |
|  | **Htt_ex1-12_Q138** | | **1d (271), 7d (469), 14d (1,196), 21d (1,944)** |  | Htt_ex1_Q91 (1d), Htt_ex1_Q91 (14d) | | <0.0001 | **** |
|  | **Htt_ex1-12_Q138+Htt_ex1_Q25** | | **1d (1), 7d (16), 14d (73), 21d (175)** |  | Htt_ex1_Q91 (1d), Htt_ex1_Q91 (21d) | | <0.0001 | **** |
|  |  | | |  | Htt_ex1_Q91 (7d), Htt_ex1_Q91 (14d) | | >0.9999 | n.s |
|  |  |  |  |  | Htt_ex1_Q91 (7d), Htt_ex1_Q91 (21d) | | 0.6464 | n.s. |
|  |  |  |  |  | Htt_ex1_Q91 (14d), Htt_ex1_Q91 (21d) | | 0.6089 | n.s. |
|  |  |  |  |  | Htt_ex1_Q91+Htt_ex1_Q25 (1d), Htt_ex1_Q91+Htt_ex1_Q25 (7d) | | >0.9999 | n.s |
|  |  |  |  |  | Htt_ex1_Q91+Htt_ex1_Q25 (1d), Htt_ex1_Q91+Htt_ex1_Q25 (14d) | | 0.9997 | n.s. |
|  |  |  |  |  | Htt_ex1_Q91+Htt_ex1_Q25 (1d), Htt_ex1_Q91+Htt_ex1_Q25 (21d) | | >0.9999 | n.s |
|  |  |  |  |  | Htt_ex1_Q91+Htt_ex1_Q25 (7d), Htt_ex1_Q91+Htt_ex1_Q25 (14d) | | >0.9999 | n.s |
|  |  |  |  |  | Htt_ex1_Q91+Htt_ex1_Q25 (7d), Htt_ex1_Q91+Htt_ex1_Q25 (21d) | | >0.9999 | n.s |
|  |  |  |  |  | Htt_ex1_Q91+Htt_ex1_Q25 (14d), Htt_ex1_Q91+Htt_ex1_Q25 (21d) | | 0.9993 | n.s. |
|  |  |  |  |  | Htt_ex1-12_Q138(1d), Htt_ex1-12_Q138 (7d) | | 0.0046 | ** |
|  |  |  |  |  | Htt_ex1-12_Q138 (1d), Htt_ex1-12_Q138 (14d) | | <0.0001 | **** |
|  |  |  |  |  | Htt_ex1-12_Q138 (1d), Htt_ex1-12_Q138 (21d) | | <0.0001 | **** |
|  |  |  |  |  | Htt_ex1-12_Q138 (7d), Htt_ex1-12_Q138 (14d) | | 0.9118 | n.s. |
|  |  |  |  |  | Htt_ex1-12_Q138 (7d), Htt_ex1-12_Q138 (21d) | | 0.0772 | n.s. |
|  |  |  |  |  | Htt_ex1-12_Q138 (14d), Htt_ex1-12_Q138 (21d) | | 0.8720 | n.s. |
|  |  |  |  |  | Htt_ex1-12_Q138+Htt_ex1_Q25 (1d), Htt_ex1-12_Q138+Htt_ex1_Q25 (7d) | | >0.9999 | n.s |
|  |  |  |  |  | Htt_ex1-12_Q138+Htt_ex1_Q25 (1d), Htt_ex1-12_Q138+Htt_ex1_Q25 (14d) | | >0.9999 | n.s |
|  |  |  |  |  | Htt_ex1-12_Q138+Htt_ex1_Q25 (1d), Htt_ex1-12_Q138+Htt_ex1_Q25 (21d) | | >0.9999 | n.s |
|  |  |  |  |  | Htt_ex1-12_Q138+Htt_ex1_Q25 (7d), Htt_ex1-12_Q138+Htt_ex1_Q25 (14d) | | >0.9999 | n.s |
|  |  |  |  |  | Htt_ex1-12_Q138+Htt_ex1_Q25 (7d), Htt_ex1-12_Q138+Htt_ex1_Q25 (21d) | | >0.9999 | n.s |
|  |  |  |  |  | Htt_ex1-12_Q138+Htt_ex1_Q25 (14d), Htt_ex1-12_Q138+Htt_ex1_Q25 (21d) | | >0.9999 | n.s |
|  |  |  |  |  | Htt_ex1_Q91, Htt_ex1_Q91+Htt_ex1_Q25 (1d) | | >0.9999 | n.s. |
|  |  |  |  |  | Htt_ex1_Q91, Htt_ex1_Q91+Htt_ex1_Q25 (7d) | | >0.9999 | n.s. |
|  |  |  |  |  | Htt_ex1_Q91, Htt_ex1_Q91+Htt_ex1_Q25 (14d) | | 0.8198 | n.s. |
|  |  |  |  |  | Htt_ex1_Q91, Htt_ex1_Q91+Htt_ex1_Q25 (21d) | | 0.0002 | ### |
|  |  |  |  |  | Htt_ex1_Q91, Htt_ex1-12_Q138 (1d) | | >0.9999 | n.s |
|  |  |  |  |  | Htt_ex1_Q91, Htt_ex1-12_Q138 (7d) | | >0.9999 | n.s |
|  |  |  |  |  | Htt_ex1_Q91, Htt_ex1-12_Q138 (14d) | | 0.7032 | n.s. |
|  |  |  |  |  | Htt_ex1_Q91, Htt_ex1-12_Q138 (21d) | | 0.0306 | ^ |
|  |  |  |  |  | Htt_ex1-12_Q138, Htt_ex1-12_Q138+Htt_ex1_Q25 (1d) | | >0.9999 | n.s |
|  |  |  |  |  | Htt_ex1-12_Q138, Htt_ex1-12_Q138+Htt_ex1_Q25 (7d) | | 0.9737 | n.s. |
|  |  |  |  |  | Htt_ex1-12_Q138, Htt_ex1-12_Q138+Htt_ex1_Q25 (14d) | | 0.0071 | ## |
|  |  |  |  |  | Htt_ex1-12_Q138, Htt_ex1-12_Q138+Htt_ex1_Q25 (21d) | | <0.0001 | #### |
|  |  |  |  |  | Htt_ex1_Q91+Htt_ex1_Q25, Htt_ex1-12_Q138+Htt_ex1_Q25 (1d) | | >0.9999 | n.s |
|  |  |  |  |  | Htt_ex1_Q91+Htt_ex1_Q25, Htt_ex1-12_Q138+Htt_ex1_Q25 (7d) | | >0.9999 | n.s |
|  |  |  |  |  | Htt_ex1_Q91+Htt_ex1_Q25, Htt_ex1-12_Q138+Htt_ex1_Q25 (14d) | | 0.6826 | n.s. |
|  |  |  |  |  | Htt_ex1_Q91+Htt_ex1_Q25, Htt_ex1-12_Q138+Htt_ex1_Q25 (21d) | | 0.9996 | n.s. |
| Fig. 2G | **Htt_ex1_Q91** | | **13,132** | unpaired t-test | Htt_ex1_Q91, Htt_ex1_Q91+Htt_ex1_Q25 | | 0.0005 | *** |
|  | **Htt_ex1_Q91+Htt_ex1_Q25** | | **754** |  |  |  |  |  |
| Fig. 2H | **Htt_ex1-12_Q138** | | **3,887** | unpaired t-test | Htt_ex1-12_Q138, Htt_ex1-12_Q138+Htt_ex1_Q25 | | <0.0001 | **** |
|  | **Htt_ex1-12_Q138+**  **Htt_ex1_Q25** | | **265** |  |  |  |  |  |
| **Figure 3** | | | | | | | | |
| Fig. 3C | **+LacZ** | | **4d (12), 10d (8)** | Two way ANOVA | 1. +LacZ (4d) 2. +shi^ts1^#1 (4d) 3. +shi^ts1^#2 (4d) | 1. +LacZ (10d) 2. +shi^ts1^#1 (10d) 3. +shi^ts1^#2 (10d) | Row (age): <0.0001 | **** |
|  |  |  |  |  |  |  | Column (genotype): 0.2112 | n.s. |
|  | **+shi^ts1^#1** | | **4d (10), 10d (8)** | Tukey’s multiple comparisons | +LacZ (4d), +shi^ts1^#1 (4d) | | 0.9687 | n.s. |
|  | **+shi^ts1^#2** | | **4d (10), 10d (11)** |  | +LacZ (4d), +shi^ts1^#2 (4d) | | 0.4043 | n.s. |
|  |  | | |  | +LacZ (10d), +shi^ts1^#1 (10d) | | 0.0419 | * |
|  |  |  |  |  | +LacZ (10d), +shi^ts1^#2 (10d) | | 0.1655 | n.s. |
| Fig. 3D | **+LacZ** | | **4d (12), 10d (8)** | Two way ANOVA | 1. +LacZ (4d) 2. +shi^ts1^#1 (4d) 3. +shi^ts1^#2 (4d) | 1. +LacZ (10d) 2. +shi^ts1^#1 (10d) 3. +shi^ts1^#2 (10d) | Row (age): <0.0001 | **** |
|  |  |  |  |  |  |  | Column (genotype): <0.0001 | **** |
|  | **+shi^ts1^#1** | | **4d (10), 10d (8)** | Tukey’s multiple comparisons | +LacZ (4d), +shi^ts1^#1 (4d) | | 0.9637 | n.s. |
|  | **+shi^ts1^#2** | | **4d (10), 10d (11)** |  | +LacZ (4d), +shi^ts1^#2 (4d) | | <0.0001 | **** |
|  |  | | |  | +LacZ (10d), +shi^ts1^#1 (10d) | | <0.0001 | **** |
|  |  |  |  |  | +LacZ (10d), +shi^ts1^#2 (10d) | | <0.0001 | **** |
| Fig. 3G | **+LacZ** | | **4d (8), 10d (10)** | Two way ANOVA | 1. +LacZ (4d) 2. +TeTxLC#1 (4d) 3. +TeTxLC#2 (4d) | 1. +LacZ (10d) 2. +TeTxLC#1 (10d) 3. +TeTxLC#2 (10d) | Row (age): <0.0001 | **** |
|  |  |  |  |  |  |  | Column (genotype): <0.0001 | **** |
|  | **+TeTxLC#1** | | **4d (10), 10d (9)** | Tukey’s multiple comparisons | +LacZ (4d), +TeTxLC#1 (4d) | | 0.0021 | ** |
|  | **+TeTxLC#2** | | **4d (10), 10d (6)** |  | +LacZ (4d), +TeTxLC#2 (4d) | | <0.0001 | **** |
|  |  | | |  | +LacZ (10d), +TeTxLC#1 (10d) | | 0.2651 | n.s. |
|  |  |  |  |  | +LacZ (10d), +TeTxLC#2 (10d) | | <0.0001 | **** |
| Fig. 3H | **+LacZ** | | **4d (8), 10d (10)** | Two way ANOVA | 1. +LacZ (4d) 2. +TeTxLC#1 (4d) 3. +TeTxLC#2 (4d) | 1. +LacZ (10d) 2. +TeTxLC#1 (10d) 3. +TeTxLC#2 (10d) | Row (age): <0.0001 | **** |
|  |  |  |  |  |  |  | Column (genotype): <0.0001 | **** |
|  | **+TeTxLC#1** | | **4d (10), 10d (9)** | Tukey’s multiple comparisons | +LacZ (4d), +TeTxLC#1 (4d) | | 0.0013 | ** |
|  | **+TeTxLC#2** | | **4d (10), 10d (6)** |  | +LacZ (4d), +TeTxLC#2 (4d) | | 0.7696 | n.s. |
|  |  | | |  | +LacZ (10d), +TeTxLC#1 (10d) | | <0.0001 | **** |
|  |  |  |  |  | +LacZ (10d), +TeTxLC#2 (10d) | | <0.0001 | **** |
| Fig. 3K | **+LacZ** | | **10** | One way ANOVA | 1. +LacZ 2. +dTrpA#1 3. +dTrpA#2 4. +dTrpA#3 | | 0.0001 | *** |
|  | **+dTrpA#1** | | **6** | Tukey’s multiple comparisons | +LacZ, +dTrpA#1 | | 0.0964 | n.s. |
|  | **+dTrpA#2** | | **11** |  | +LacZ, +dTrpA#2 | | 0.0008 | *** |
|  | **+dTrpA#3** | | **10** |  | +LacZ, +dTrpA#3 | | <0.0001 | **** |
| Fig. 3L | **+LacZ** | | **10** | One way ANOVA | 1. +LacZ 2. +dTrpA#1 3. +dTrpA#2 4. +dTrpA#3 | | <0.0001 | **** |
|  | **+dTrpA#1** | | **6** | Tukey’s multiple comparisons | +LacZ, +dTrpA#1 | | <0.0001 | **** |
|  | **+dTrpA#2** | | **11** |  | +LacZ, +dTrpA#2 | | 0.0033 | ** |
|  | **+dTrpA#3** | | **10** |  | +LacZ, +dTrpA#3 | | <0.0001 | **** |
| **Figure 4** | | | | | | | | |
| Fig. 4C | **+LacZ** | | **10** | unpaired t-test | +LacZ, +shi^ts1^ (intensity) | | 0.0002 | *** |
|  | **+shi^ts1^** | | **8** | unpaired t-test | +LacZ, +shi^ts1^ (volume) | | <0.0001 | **** |
| Fig. 4F | **+LacZ** | | **9** | One way ANOVA | 1. +LacZ 2. +shi^ts1^#1 3. +shi^ts1^#2 | | 0.0908 | n.s. |
|  | **+shi^ts1^#1** | | **8** | Tukey’s multiple comparisons | +LacZ, +shi^ts1^#1 | | 0.0773 | n.s. |
|  | **+shi^ts1^#2** | | **10** |  | +LacZ, +shi^ts1^#2 | | 0.6574 | n.s. |
| Fig. 4G | **+LacZ** | | **9** | One way ANOVA | 1. +LacZ 2. +shi^ts1^#1 3. +shi^ts1^#2 | | <0.0001 | **** |
|  | **+shi^ts1^#1** | | **8** | Tukey’s multiple comparisons | +LacZ, +shi^ts1^#1 | | <0.0001 | **** |
|  | **+shi^ts1^#2** | | **10** |  | +LacZ, +shi^ts1^#2 | | 0.0183 | * |
| **Figure 5** | | | | | | | | |
| Fig. 5C | ***drpr ^+/-^* females** | | **7d (12), 13d (20)** | Two way ANOVA | 1. *drpr ^+/-^* females (7d) 2. *drpr ^-/-^* females (7d) 3. *drpr ^+/-^* males (7d) 4. *drpr ^-/-^* males (7d) | 1. *drpr ^+/-^* females (13d) 2. *drpr ^-/-^* females (13d) | Row (age): 0.0002 | *** |
|  |  |  |  |  |  |  | Column (genotype): <0.0001 | **** |
|  | ***drpr ^-/-^* females** | | **7d (10), 13d (13)** | Tukey’s multiple comparisons | *drpr ^+/-^* females (7d); *drpr ^-/-^* females (7d) | | 0.9368 | n.s. |
|  | ***drpr ^+/-^* males** | | **7d (18)** |  | *drpr ^+/-^* males (7d); *drpr ^-/-^* males (7d) | | 0.1553 | n.s. |
|  | ***drpr ^-/-^* males** | | **7d (6)** |  | *drpr ^+/-^* females (13d); *drpr ^-/-^* females (13d) | | 0.1591 | n.s. |
| Fig. 5D | ***drpr ^+/-^* females** | | **7d (12), 13d (20)** | Two way ANOVA | 1. *drpr ^+/-^* females (7d) 2. *drpr ^-/-^* females (7d) 3. *drpr ^+/-^* males (7d) 4. *drpr ^-/-^* males (7d) | 1. *drpr ^+/-^* females (13d) 2. *drpr ^-/-^* females (13d) | Row (age): 0.9654 | n.s. |
|  |  |  |  |  |  |  | Column (genotype): <0.0001 | **** |
|  | ***drpr ^-/-^* females** | | **7d (10), 13d (13)** | Tukey’s multiple comparisons | *drpr ^+/-^* females (7d); *drpr ^-/-^* females (7d) | | 0.0188 | * |
|  | ***drpr ^+/-^* males** | | **7d (18)** |  | *drpr ^+/-^* males (7d); *drpr ^-/-^* males (7d) | | 0.0014 | ** |
|  | ***drpr ^-/-^* males** | | **7d (6)** |  | *drpr ^+/-^* females (13d); *drpr ^-/-^* females (13d) | | <0.0001 | **** |
| Fig. 5G | ***drpr ^+/+^*** | | **1,556** | Unpaired t-test | *drpr ^+/+^*; *drpr ^-/-^* | | <0.0001 | **** |
|  | ***drpr ^-/-^*** | | **1,749** |  |  |  |  |  |
| **Figure 5 - figure supplement 1** | | | | | | | | |
| Fig. 5 – fig supp 1C | **+shi^ts1^, *drpr ^+/+^*** | | **11** | Unpaired t-test | +shi^ts1^, *drpr ^+/+^*; +shi^ts1^, *drpr ^-/-^* | | 0.6555 | n.s. |
|  | **+shi^ts1^, *drpr ^-/-^*** | | **8** |  |  |  |  |  |
| Fig. 5 – fig supp 1D | **+shi^ts1^, *drpr ^+/+^*** | | **11** | Unpaired t-test | +shi^ts1^, *drpr ^+/+^*; +shi^ts1^, *drpr ^-/-^* | | <0.0001 | **** |
|  | **+shi^ts1^, *drpr ^-/-^*** | | **8** |  |  |  |  |  |
| **Figure 5 - figure supplement 2** | | | | | | | | |
| Fig. 5 – fig supp 2E | **control** | | **5** | One way ANOVA | 1. control 2. +repo-Gal80 3. +FFLuc 4. +Drpr^RNAi^ | | 0.0258 | * |
|  | **+repo-Gal80** | | **6** | Tukey’s multiple comparisons | control; +repo-Gal80 | | 0.2665 | n.s. |
|  | **+FFLuc** | | **11** |  | +FFLuc; +Drpr^RNAi^ | | 0.0524 | n.s. |
|  | **+Drpr^RNAi^** | | **6** |  | | | | |
| Fig. 5 – fig supp 2F | **control** | | **5** | One way ANOVA | 1. control 2. +repo-Gal80 3. +FFLuc 4. +Drpr^RNAi^ | | 0.0139 | * |
|  | **+repo-Gal80** | | **6** | Tukey’s multiple comparisons | control; +repo-Gal80 | | 0.4347 | n.s. |
|  | **+FFLuc** | | **11** |  | +FFLuc; +Drpr^RNAi^ | | 0.5493 | n.s. |
|  | **+Drpr^RNAi^** | | **6** |  | | | | |
| **Figure 5 - figure supplement 3** | | | | | | | | |
| Fig. 5 – fig supp 3E | **Atg8a** | | **12** | One way ANOVA | 1. Atg8a 2. Lamp1 3. mCD8 | | 0.1024 | n.s. |
|  | **Lamp1** | | **12** | Tukey’s multiple comparisons | Atg8a; mCD8 | | 0.6955 | n.s. |
|  | **mCD8** | | **10** |  | Lamp1; mCD8 | | 0.4165 | n.s. |
| Fig. 5 – fig supp 3F | **Atg8a** | | **12** | One way ANOVA | 1. Atg8a 2. Lamp1 3. mCD8 | | 0.0008 | *** |
|  | **Lamp1** | | **12** | Tukey’s multiple comparisons | Atg8a; mCD8 | | 0.1617 | n.s. |
|  | **mCD8** | | **10** |  | Lamp1; mCD8 | | 0.0508 | n.s. |
| **Figure 6** | | | | | | | | |
| Fig. 6E | **Htt_ex1_Q25, *drpr ^+/-^*** | | **6** | One way ANOVA | 1. Htt_ex1_Q25*, drpr ^+/-^* 2. Htt_ex1_Q91, *drpr ^+/-^* 3. Htt_ex1_Q25, *drpr ^-/-^* 4. Htt_ex1_Q91, *drpr ^-/-^* | | <0.0001 | **** |
|  | **Htt_ex1_Q91, *drpr ^+/-^*** | | **7** | Tukey’s multiple comparisons | Htt_ex1_Q25, *drpr ^+/-^*; Htt_ex1_Q91, *drpr ^+/-^* | | 0.1584 | n.s. |
|  | **Htt_ex1_Q25, *drpr ^-/-^*** | | **6** |  | Htt_ex1_Q25, *drpr ^-/-^*; Htt_ex1_Q91, *drpr ^-/-^* | | 0.0003 | *** |
|  | **Htt_ex1_Q91, *drpr ^-/-^*** | | **8** |  | Htt_ex1_Q25, *drpr ^+/-^*; Htt_ex1_Q25, *drpr ^-/-^* | | 0.6955 | n.s. |
|  |  | | |  | Htt_ex1_Q91, *drpr ^+/-^*; Htt_ex1_Q91, *drpr ^-/-^* | | 0.0031 | ** |
| Fig. 6J | **+LacZ in ORNs** | | **8** | One way ANOVA | 1. +LacZ in ORNs 2. +p35 in ORNs 3. +LacZ in PNs 4. +p35 in PNs | | 0.0160 | * |
|  | **+p35 in ORNs** | | **8** | Tukey’s multiple comparisons | +LacZ in ORNs; +p35 in ORNs | | 0.9424 | n.s. |
|  | **+LacZ in PNs** | | **7** |  | +LacZ in PNs; +p35 in PNs | | 0.0799 | n.s. |
|  | **+p35 in PNs** | | **7** |  | | | | |
| Fig. 6K | **+LacZ in ORNs** | | **8** | One way ANOVA | 1. +LacZ in ORNs 2. +p35 in ORNs 3. +LacZ in PNs 4. +p35 in PNs | | 0.0055 | ** |
|  | **+p35 in ORNs** | | **8** | Tukey’s multiple comparisons | +LacZ in ORNs; +p35 in ORNs | | 0.0292 | * |
|  | **+LacZ in PNs** | | **7** |  | +LacZ in PNs; +p35 in PNs | | 0.9129 | n.s. |
|  | **+p35 in PNs** | | **7** |  | | | | |
| **Figure 7** | | | | | | | | |
| Fig. 7I | **control** | | **0d (12), 1d (10), 2d (12), 3d (10), 4d (11), 5d (10), 7d (14)** | One way ANOVA | - 1. control (0d, 1d, 2d, 3d, 4d, 5d, 7d)   8. +Drpr^RNAi^ (7d) | | <0.0001 | **** |
|  | **+Drpr^RNAi^** | | **7d (8)** | Tukey’s multiple comparisons | control (0d); control (1d) | | 0.1603 | n.s. |
|  |  | |  |  | control (0d); control (2d) | | <0.0001 | **** |
|  |  | |  |  | control (0d); control (3d) | | <0.0001 | **** |
|  |  | |  |  | control (0d); control (4d) | | <0.0001 | **** |
|  |  | |  |  | control (0d); control (5d) | | <0.0001 | **** |
|  |  | |  |  | control (0d); control (7d) | | <0.0001 | **** |
|  |  | |  |  | control (1d); control (2d) | | 0.3062 | n.s. |
|  |  | |  |  | control (1d); control (3d) | | 0.0008 | *** |
|  |  | |  |  | control (1d); control (4d) | | 0.0004 | *** |
|  |  | |  |  | control (1d); control (5d) | | 0.1532 | n.s. |
|  |  | |  |  | control (1d); control (7d) | | 0.0007 | *** |
|  |  | |  |  | control (2d); control (3d) | | 0.4286 | n.s. |
|  |  | |  |  | control (2d); control (4d) | | 0.3373 | n.s. |
|  |  | |  |  | control (2d); control (5d) | | >0.9999 | n.s. |
|  |  | |  |  | control (2d); control (7d) | | 0.5489 | n.s. |
|  |  | |  |  | control (3d); control (4d) | | >0.9999 | n.s. |
|  |  | |  |  | control (3d); control (5d) | | 0.7851 | n.s. |
|  |  | |  |  | control (3d); control (7d) | | >0.9999 | n.s. |
|  |  | |  |  | control (4d); control (5d) | | 0.7090 | n.s. |
|  |  | |  |  | control (4d); control (7d) | | >0.9999 | n.s. |
|  |  | |  |  | control (5d); control (7d) | | 0.8919 | n.s. |
|  |  | |  |  | control (7d); +Drpr^RNAi^ (7d) | | <0.0001 | #### |
| Fig. 7J | **control** | | **0d (12), 1d (10), 2d (12), 3d (10), 4d (11), 5d (10), 7d (14)** | One way ANOVA | - 1. control (0d, 1d, 2d, 3d, 4d, 5d, 7d)   8. +Drpr^RNAi^ (7d) | | <0.0001 | **** |
|  | **+Drpr^RNAi^** | | **7d (8)** | Tukey’s multiple comparisons | mCherry+/3xHA+: control (0d); control (1d) | | >0.9999 | n.s. |
|  |  | | |  | mCherry+/3xHA+: control (0d); control (2d) | | 0.9562 | n.s. |
|  |  |  |  |  | mCherry+/3xHA+: control (0d); control (3d) | | 0.0004 | *** |
|  |  |  |  |  | mCherry+/3xHA+: control (0d); control (4d) | | 0.0535 | n.s. |
|  |  |  |  |  | mCherry+/3xHA+: control (0d); control (5d) | | <0.0001 | **** |
|  |  |  |  |  | mCherry+/3xHA+: control (0d); control (7d) | | 0.0001 | **** |
|  |  |  |  |  | mCherry+/3xHA+: control (1d); control (2d) | | 0.9496 | n.s. |
|  |  |  |  |  | mCherry+/3xHA+: control (1d); control (3d) | | 0.0006 | *** |
|  |  |  |  |  | mCherry+/3xHA+: control (1d); control (4d) | | 0.0627 | n.s. |
|  |  |  |  |  | mCherry+/3xHA+: control (1d); control (5d) | | <0.0001 | **** |
|  |  |  |  |  | mCherry+/3xHA+: control (1d); control (7d) | | 0.0002 | *** |
|  |  |  |  |  | mCherry+/3xHA+: control (2d); control (3d) | | 0.0119 | * |
|  |  |  |  |  | mCherry+/3xHA+: control (2d); control (4d) | | 0.4239 | n.s. |
|  |  |  |  |  | mCherry+/3xHA+: control (2d); control (5d) | | <0.0001 | **** |
|  |  |  |  |  | mCherry+/3xHA+: control (2d); control (7d) | | 0.0065 | ** |
|  |  |  |  |  | mCherry+/3xHA+: control (3d); control (4d) | | 0.7531 | n.s. |
|  |  |  |  |  | mCherry+/3xHA+: control (3d); control (5d) | | 0.6539 | n.s. |
|  |  |  |  |  | mCherry+/3xHA+: control (3d); control (7d) | | >0.9999 | n.s. |
|  |  |  |  |  | mCherry+/3xHA+: control (4d); control (5d) | | 0.0293 | * |
|  |  |  |  |  | mCherry+/3xHA+: control (4d); control (7d) | | 0.7520 | n.s. |
|  |  |  |  |  | mCherry+/3xHA+: control (5d); control (7d) | | 0.4856 | n.s. |
|  |  |  |  |  | mCherry+/3xHA+: control (7d); +Drpr^RNAi^ (7d) | | 0.0039 | ## |
|  |  |  |  |  | mCherry+/3xHA+/YFP+: control (0d); control (1d) | | >0.9999 | n.s. |
|  |  |  |  |  | mCherry+/3xHA+/YFP+: control (0d); control (2d) | | 0.9986 | n.s. |
|  |  |  |  |  | mCherry+/3xHA+/YFP+: control (0d); control (3d) | | 0.0070 | ** |
|  |  |  |  |  | mCherry+/3xHA+/YFP+: control (0d); control (4d) | | <0.0001 | **** |
|  |  |  |  |  | mCherry+/3xHA+/YFP+: control (0d); control (5d) | | <0.0001 | **** |
|  |  |  |  |  | mCherry+/3xHA+/YFP+: control (0d); control (7d) | | <0.0001 | **** |
|  |  |  |  |  | mCherry+/3xHA+/YFP+: control (1d); control (2d) | | 0.9990 | n.s. |
|  |  |  |  |  | mCherry+/3xHA+/YFP+: control (1d); control (3d) | | 0.0117 | * |
|  |  |  |  |  | mCherry+/3xHA+/YFP+: control (1d); control (4d) | | <0.0001 | **** |
|  |  |  |  |  | mCherry+/3xHA+/YFP+: control (1d); control (5d) | | <0.0001 | **** |
|  |  |  |  |  | mCherry+/3xHA+/YFP+: control (1d); control (7d) | | <0.0001 | **** |
|  |  |  |  |  | mCherry+/3xHA+/YFP+: control (2d); control (3d) | | 0.0332 | * |
|  |  |  |  |  | mCherry+/3xHA+/YFP+: control (2d); control (4d) | | <0.0001 | **** |
|  |  |  |  |  | mCherry+/3xHA+/YFP+: control (2d); control (5d) | | <0.0001 | **** |
|  |  |  |  |  | mCherry+/3xHA+/YFP+: control (2d); control (7d) | | <0.0001 | **** |
|  |  |  |  |  | mCherry+/3xHA+/YFP+: control (3d); control (4d) | | 0.0003 | *** |
|  |  |  |  |  | mCherry+/3xHA+/YFP+: control (3d); control (5d) | | <0.0001 | **** |
|  |  |  |  |  | mCherry+/3xHA+/YFP+: control (3d); control (7d) | | <0.0001 | **** |
|  |  |  |  |  | mCherry+/3xHA+/YFP+: control (4d); control (5d) | | 0.1420 | n.s. |
|  |  |  |  |  | mCherry+/3xHA+/YFP+: control (4d); control (7d) | | 0.7181 | n.s. |
|  |  |  |  |  | mCherry+/3xHA+/YFP+: control (5d); control (7d) | | 0.8902 | n.s. |
|  |  |  |  |  | mCherry+/3xHA+/YFP+: control (7d); +Drpr^RNAi^ (7d) | | <0.0001 | #### |
